# Development of a novel high resolution melting assay for identification and differentiation of all known 19 serovars of *Actinobacillus pleuropneumoniae*

**DOI:** 10.1002/mbo3.1272

**Published:** 2022-03-16

**Authors:** Simone Scherrer, Sophie Peterhans, Christine Neupert, Fenja Rademacher, Giody Bartolomei, Xaver Sidler, Roger Stephan

**Affiliations:** ^1^ Institute for Food Safety and Hygiene, Section of Veterinary Bacteriology, Vetsuisse Faculty University of Zurich Zurich Switzerland; ^2^ Malcisbo AG Schlieren Switzerland; ^3^ Department of Farm Animals, Division of Swine Medicine, Vetsuisse Faculty University of Zurich Zurich Switzerland

**Keywords:** *Actinobacillus pleuropneumoniae*, capsule typing, high resolution melting, serovar

## Abstract

*Actinobacillus pleuropneumoniae* is the etiological agent of porcine pleuropneumonia, a respiratory infectious disease responsible for global economic losses in the pig industry. From a monitoring perspective as well as due to the different courses of disease associated with the various serovars, it is essential to distinguish them in different herds or countries. In this study, we developed a novel high resolution melting (HRM) assay based on reference strains for each of the 19 known serovars and additional 15 clinical *A. pleuropneumoniae* isolates. The novel HRM comprises the species‐specific APP‐HRM1 and two serovar‐specific HRM assays (APP‐HRM2 and APP‐HRM3). APP‐HRM1 allowed polymerase chain reaction (PCR) amplification of *apxIV* resulting in an *A. pleuropneumoniae* specific melting curve, while *nadV* specific primers differentiated biovar 2 from biovar 1 isolates. Using APP‐HRM2 and APP‐HRM3, 13 *A. pleuropneumoniae* serovars can be determined by inspecting the assigned melting temperature. In contrast, serovar 3 and 14, serovar 9 and 11, and serovar 5 and 15 have partly overlapping melting temperatures and thus represent a challenge to accurately distinguish them. Consequently, to unambiguously ensure the correct assignment of the serovar, it is recommended to perform the serotyping HRM assay using a positive control for each serovar. This rapid and user‐friendly assay showed high sensitivity with 1.25 fg–125 pg of input DNA and a specificity of 100% to identify *A. pleuropneumoniae*. Characteristic melting patterns of amplicons might allow detecting new serovars. The novel HRM assay has the potential to be implemented in diagnostic laboratories for better surveillance of this pathogen.

## Graphical abstract

*Actinobacillus pleuropneumoniae* infection is a worldwide problem in the pig industry. It is important to differentiate between the 19 serovars using an efficient molecular tool, thereby improving presently available diagnostic methods. Knowing the prevalent serovar, the right vaccines could be administered and pigs potentially carrying virulent strains could be prevented from being introduced into farms.

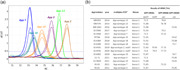

## 1 INTRODUCTION

*Actinobacillus pleuropneumoniae* is the etiological agent of porcine pleuropneumonia, a respiratory infectious disease responsible for global economic losses due to high mortality rates and high treatment costs (Gottschalk & Broes, 2019). *A. pleuropneumoniae* isolates can be divided into biovar I and biovar II, requiring exogenous nicotinamide adenine dinucleotide (NAD) for growth in the case of biovar I, whereas biovar II strains comprise *nadV* responsible for NAD‐independent growth (Pohl et al., 1983). Various serovars associated with different courses of disease are described, which can be differentiated by the expression of several capsular antigens (Sassu et al., 2018). Currently, there are 19 recognized serovars of *A. pleuropneumoniae* based on the composition of the capsular polysaccharide (CPS) (Bossé et al., 2018; Stringer et al., 2021).

The pore‐forming exotoxins ApxI, ApxII, and ApxIII are important virulence factors of *A. pleuropneumoniae*. All virulent strains express one or two of these toxins. ApxIV toxin is essential for full virulence of *A. pleuropneumoniae*. It is expressed by all isolates of this species making *apxIV* a useful species‐specific marker (Chiers et al., 2010; Frey, 1995; Schaller et al., 1999).

Serotyping of *A. pleuropneumoniae* has formerly been performed serologically. However, due to highly similar capsular and lipopolysaccharide O‐antigen epitopes between certain serovars, cross‐reactions have been observed leading to incorrect serotyping results (Gottschalk, 2015; Mittal, 1990; Mittal & Bourdon, 1991; Mittal et al., 1988). Presently, many laboratories use multiplex polymerase chain reaction (PCR) assays for serotyping purposes (Bossé et al., 2018; Stringer et al., 2021). However, using conventional PCR assays is more time‐consuming and it remains a challenge to assign the correct amplicon size.

High resolution melting (HRM) is a rapid and low‐cost PCR‐based method characterizing PCR amplicons according to their dissociation behavior. Once a PCR reaction has been completed, a stepwise increase of temperature results in dissociation of the double‐stranded DNA into single strands leading to a decrease in fluorescence intensity. The dissociation of the double‐stranded DNA is dependent on the sequence of the amplicon, GC content, and length, therefore, contributing to a specific melting temperature for each amplicon (Vossen et al., 2009).

Serovar classification of *A. pleuropneumoniae* isolates helps trace certain serovars that cause severe diseases on a farm allowing epidemiological surveillance and is useful to provide information for vaccine development (Gottschalk, 2012). In this study, we propose a novel HRM assay to simultaneously identify *A. pleuropneumoniae* and its biovar based on *apxIV* and *nadV* on one hand and differentiate all known 19 serovars using CPS cluster as a target region on the other hand.

## 2 MATERIALS AND METHODS

### 2.1 Bacterial strains and clinical isolates

For the development of the novel HRM assay, the following *A. pleuropneumoniae* reference strains of serovars 1–18 were used: ATCC 27088, P1875, ORG1224, M62, K17, L20, femø, WF83, 405, CVJ 13261, D13039, 56153, 8329, N 273, 3906, HS143, A‐85/14, 16287‐1, and 7311555 (Table 1). *A. pleuropneumoniae* G1 9626 (serovar 19) was isolated and sequenced at the Section of Veterinary Bacteriology, University of Zurich (Peterhans et al., 2021) and was included as a serovar 19 reference strain (Table 1). Fifteen clinical isolates (Table 2), obtained from routine diagnostic submissions to the Section of Veterinary Bacteriology, the University of Zurich between 2012 and 2021, were tested in the HRM assay. Clinical samples were cultured on blood and chocolate agar plates (Thermo Fisher Diagnostics AG) for 24–48 h at 37°C in 5% CO_2_ to get clinical isolates. Biovar I or biovar II was confirmed phenotypically depending on whether growth on blood agar plates was observed (biovar II) or not (biovar I).

**Table 1:** *Actinobacillus pleuropneumoniae* reference strains used for the development of the high resolution melting (HRM) assays

Strain	Serovar	Biovar	Source/reference
*A. pleuropneumoniae* ATCC 27088	1	1	ATCC
*A. pleuropneumoniae* P1875	2	1	Veterinary Bacteriology, Vetsuisse Faculty, Bern, Switzerland
*A. pleuropneumoniae* ORG1224	3	1	Veterinary Bacteriology, Vetsuisse Faculty, Bern, Switzerland
*A. pleuropneumoniae* M62	4	1	Department of Microbiology, Royal Dental College, Aarhus, Denmark
*A. pleuropneumoniae* K17	5a	1	Department of Microbiology, Royal Dental College, Aarhus, Denmark
*A. pleuropneumoniae* L20	5b	1	Department of Microbiology, Royal Dental College, Aarhus, Denmark
*A. pleuropneumoniae* femø	6	1	Department of Microbiology, Royal Dental College, Aarhus, Denmark
*A. pleuropneumoniae* WF83	7	1	Department of Veterinary Microbiology and Immunology, University of Guelph, Ontario, Canada
*A. pleuropneumoniae* 405	8	1	Danish Veterinary Laboratory, Copenhagen, Denmark
*A. pleuropneumoniae* CVI 13261	9	1	Danish Veterinary Laboratory, Copenhagen, Denmark
*A. pleuropneumoniae* D13039	10	1	Danish Veterinary Laboratory, Copenhagen, Denmark
*A. pleuropneumoniae* 56153	11	1	Department of Bacteriology, Central Veterinary Institute, Lelystad, The Netherlands
*A. pleuropneumoniae* 8329	12	1	Danish Veterinary Laboratory, Copenhagen, Denmark
*A. pleuropneumoniae* N 273	13	2	Department of Epizootiology, University of Veterinary Science, Budapest, Hungary
*A. pleuropneumoniae* 3906	14	2	Danish Veterinary Laboratory, Copenhagen, Denmark
*A. pleuropneumoniae* HS143	15	1	Department of Primary Industries Queensland, Animal Research Institute, Yeerongpilly, Australia
*A. pleuropneumoniae* A‐85/14	16	1	Department of Infectious Disease, Imperial College, London, United Kingdom
*A. pleuropneumoniae* 16287‐1	17	1	Department of Infectious Disease, Imperial College, London, United Kingdom
*A. pleuropneumoniae* 7311555	18	1	Department of Infectious Disease, Imperial College, London, United Kingdom
*A. pleuropneumoniae* G1 9626	19	1	Veterinary Bacteriology, Vetsuisse Faculty, Zurich, Switzerland

**Table 2:** Clinical isolates of *Actinobacillus pleuropneumoniae* used in the study

*A. pleuropneumoniae* isolates	Year	Multiplex PCR^a^	Biovar	Origin	Anamnesis/clinical symptoms
MB 893	2014	*A. pleuropneumoniae* serovar 13	Biovar 1	Joint	Lameness, diarrhea, decreased growth rate
MB 976	2014	*A. pleuropneumoniae* serovar 13	Biovar 1	Wound	Neurological symptoms
MB 1465	2016	*A. pleuropneumoniae* serovar 18	Biovar 1	Lung	Sudden death
SS 3906	2017	*A. pleuropneumoniae* serovar 7	Biovar 1	Lung	Pneumonia
SS 3948	2017	*A. pleuropneumoniae* serovar 7	Biovar 1	Lung	Diarrhea, decreased growth rate, sneezing
PP766	2018	*A. pleuropneumoniae* serovar 19	Biovar 1	Lung	Lung lesions
SS 4384	2018	*A. pleuropneumoniae* serovar 18	Biovar 1	Lung	Diarrhea, pneumonia
SS 4388	2019	*A. pleuropneumoniae* serovar 18	Biovar 1	Lung	Pneumonia
SS 4935	2020	*A. pleuropneumoniae* serovar 2, *nadV*	Biovar 2	Lung	Lung lesions
SS 4936	2020	*A. pleuropneumoniae* serovar 2, *nadV*	Biovar 2	Lung	Lung lesions
SS 4983	2020	*A. pleuropneumoniae* serovar 19	Biovar 1	Lung	Pneumonia, sudden death, lung lesions
21‐71	2021	*A. pleuropneumoniae* serovar 3	Biovar 1	Joint	Swollen joints
G1 9669	2021	*A. pleuropneumoniae* serovar 19	Biovar 1	Lung	Pneumonia, sudden death
XS‐03	2021	A. pleuropneumoniae serovar 7	Biovar 1	Lung	Unknown
RS‐01	2021	A. pleuropneumoniae serovar 3	Biovar 1	Lung	Pneumonia

Abbreviation: PCR, polymerase chain reaction.

^a^ Serovar characterization by multiplex PCR (Bossé et al., 2018; Stringer et al., 2021).

In addition, 27 nontarget isolates comprising 18 different bacterial strains were tested as negative controls. These included *A. minor* (*n* = 1), *A. suis* (*n* = 1), *Pasteurella multocida* (*n* = 3), *Glaesserella parasuis* (*n* = 1), *Bordetella bronchiseptica* (*n* = 2), *Streptococcus suis* (*n* = 5), *Staphylococcus aureus* (*n* = 1), *Staphylococcus hyicus* (*n* = 2), *Staphylococcus chromogenes* (*n* = 1), *Erysipelothrix rhusiopathiae* (*n* = 1), *Trueperella pyogenes* (*n* = 1), *A. rossi* (*n* = 1), *A. seminis* (*n* = 1), *Yersinia enterocolitica* (*n* = 1), *Pseudomonas aeruginosa* (*n* = 1), *Escherichia coli* (*n* = 2), *Enteroccoccus faecalis* (*n* = 1), and *Rhodococcus hoagii* (*n* = 1).

### 2.2 Identification of clinical isolates

Serovars of clinical isolates were first identified using multiplex PCR of published protocols (Bossé et al., 2018; Stringer et al., 2021). Briefly, as proposed, two PCR reactions were performed using Qiagen HotStart Taq DNA Polymerase (Qiagen) in a minimal total reaction volume of 10 µl including primers at a final concentration of 0.3 μM each and 1 µl of DNA template. PCR cycling was performed with initial activation of Taq Polymerase for 15 min, followed by 35 cycles at 30 s for 94°C for denaturation, 90 s at 60°C, for annealing and 150 s at 72°C for elongation followed by a final extension step of 10 min at 72°C. Size analysis of PCR products was performed on a capillary electrophoresis QIAxcel Advanced device (Qiagen) using a screening cartridge, QX 15 bp–3 kb alignment marker, and QX 100 bp–2.5 kb size marker (Qiagen) according to the manufacturer's instructions. The resulting electropherograms were inspected with the QIAxcel ScreenGel 1.2.0 software (Qiagen).

### 2.3 HRM development and optimization

Primers were designed using CLC Main Workbench software 7.5.1 (Qiagen) with CPS sequences of *A. pleuropneumoniae* retrieved from the NCBI databank targeting the same CPS loci as described previously (Stringer et al., 2021). The specificity of primer sequences was confirmed by BLAST searches. Oligonucleotide primers were synthesized by Microsynth (Balgach, Switzerland). All HRM experiments were performed on a Rotor‐Gene Q (Qiagen) using Type‐it HRM PCR Kit (Qiagen) with a total reaction volume of 15 µl. One microliter of sample DNA was added to a reaction mixture containing Type‐it HRM PCR Master Mix (2x) (Qiagen), primers at a concentration indicated (Tables 3, 4, 5) targeting capsular gene regions of different serovars of *A. pleuropneumoniae* and ultrapure water. The PCR thermocycling conditions were as follows: initial denaturation at 95°C for 5 min, 40 cycles with denaturation at 95°C for 10 s, and annealing/extension at 55°C (APP‐HRM1) and 62°C (APP‐HRM 2 and APP‐HRM3), respectively, for 30 s followed by a final cycling step for 10 s at 95°C and 2 min at 40°C. Finally, an HRM ramping from 62–95°C with fluorescence data acquisition at 0.1°C increments every 2 s was performed to generate *A. pleuropneumoniae* serovar‐specific melting curves. DNA originating from reference strains was used as positive controls in each PCR run. To exclude contaminations in the reaction mixture, ultrapure water was added as a negative control in each experiment.

**Table 3:** APP‐HRM 1 primers for detection of *Actinobacillus pleuropneumoniae* and biovar 2

Primer name	Sequence 5ʹ–3ʹ	Target gene	Reference	Amplicon size (bp)	Final concentration PCR (nM)	Amplicon melting temperature (*T*_m_) HRM
apxIVHRM_for	CCGAGAAAATAACGATTTG	*apxIV*	This study	77	1066	71.8 ± 0.2
apxIVHRM_rev	GGTGTGAATACCAATTTTG	*axpIV*	This study		1066	
nadVHRM_for	CAATGCGAGGAATGAGTTCTT	*nadV*	This study	155	150	79.8 ± 0.2
nadVHRM_rev	TTCGGAGGCAGGAATAGAC	*nadV*	This study		150	

Abbreviations: APP‐HRM, *Actinobacillus pleuropneumoniae*‐high resolution melting; PCR, polymerase chain reaction.

**Table 4:** APP‐HRM2 primers for detection of *Actinobacillus pleuropneumoniae* serovars 1, 2, 4, 5, 7, 8, 10, 13, and 15

Primer name	Sequence 5ʹ–3ʹ	Target gene	References	Amplicon size (bp)	Final concentration PCR (nM)	Amplicon melting temperature (*T*_m_) HRM
APP1HRM_for	GAAAATGCAAGTACTACTAGCTTCTCCT	*cps1B*	This study	169	400	75.4 ± 0.1
APPP1HRM_rev	GGCATTAGCTTTTAATGATAATACTAGTAATTGTTC	*cps1B*	This study		400	
APP2HRM_for	ACCAGAACGTCCTTCTAAAGC	*cps2D*	This study	165	250	77.6 ± 0.1
APP2HRM_rev	CTAAGAGCGAATCCATTCCCAT	*cps2D*	This study		250	
APP4HRM_for	TGGGTTTGGTCCTGTTGTG	*cps4B*	This study	199	200	76.3 ± 0.1
AP4R	GGCTTTCTCCGTGTATGAATAAAGTG	*cps4B*	Bossé et al. (2018)		200	
APP5HRM_for	AGCCACAAGACCCGAATG	*cps5B*	This study	118	400	74.5 ± 0.1
APP5HRM_rev	AATACCAAGCAGCAGCCAT	*cps5B*	This study		400	
AP7F	TTGGAATGGATTCATGATTGGGC	*cps7E*	Bossé et al. (2014)	191	200	73.1 ± 0.2
APP7HRM_rev	CAAGGTTTCCCTTGAGGACCAT	*cps7E*	This study		200	
APP8HRM_for	TGTTATTTAGGCAGTTCTGGAGAAC	*cps8G*	This study	114	300	73.7 ± 0.1
APP8HRM_rev	AGCTCCAAGAAGAGTACAATCATCT	*cps8G*	This study		300	
APP10HRM_for	GTCTGGTGGTGATGGAACAAG	*cps10A*	This study	180	400	77.3 ± 0.1
APP10HRM_rev	TGATGCGAAATAGTAGATTGGTGCT	*cps10A*	This study		400	
AP13F	GTTGTGTATCGAGGTTGGCATTTC	*cps13E*	Bossé et al. (2018)	169	250	76.8 ± 0.1
APP13HRM_rev	TCTTTATCTAATTCACTTGCTAGGTGTTC	*cps13E*	This study		250	
APP15HRM_for	AGTATTATTAAGTGGCTTACCAAGACA	*cps15B*	This study	166	500	74.8 ± 0.2
APP15HRM_rev	TGAAGATAATAACTCTACCCAATTTCGT	*cps15B*	This study		500	

Abbreviations: APP‐HRM, *Actinobacillus pleuropneumoniae*‐high resolution melting; PCR, polymerase chain reaction.

**Table 5:** APP‐HRM3 primers for detection of *Actinobacillus pleuropneumoniae* serovars 3, 6, 9, 11, 12, 14, 16, 17, 18, and 19

Primer name	Sequence 5ʹ–3ʹ	Target gene	References	Amplicon size (bp)	Final concentration PCR (nM)	Amplicon melting temperature (*T*_m_) HRM
APP3HRM_for	ACACATATCAATCGGCAGGAGT	*cps3F*	This study	141	200	75.7 ± 0.2
AP3R	CATTCGCACCAGCAATCACC	*cps3F*	Bossé et al. (2018)		200	
APP6HRM_for	CTCAATGCTATCATGCTCAACAAATG	*cps6F*	This study	200	200	77.6 ± 0.1
AP6R	GTCTGAAGTTTTATTCGCAGCTCC	*cps6F*	Bossé et al. (2018)		200	
APP9/11HRM_for	CTTTACTTGAACCTAGGGTTAAGTTTATC	cps9/11F11F	This study	85	500	73.3 ± 0.1/73.4 ± 0.1
APP9/11HRM_rev	GCCTTATCACCTAATAGCACTGAG	cps9/11F11F	This study		500	
AP12F	TAAAGGTATTATAACGCCGGCTCT	*cps12A*	Bossé et al. (2014)	169	200	77.2 ± 0.1
APP12HRM_rev	TCTCATAACGCAGCCATGC	*cps12A*	This study		200	
APP14HRM_for	TCTACGGAAACCAAAGCTATGATT	*cps14G*	This study	149	500	75.5 ± 0.1
APP14HRM_rev	TGCTTCCAAGCGAGAATCA	*cps14G*	This study		500	
AP16F	TTACTCACTTGGGCTAGGGATAG	*cps16C*	Bossé et al. (2017)	125	400	76.4 ± 0.1
APP16HRM_rev	TGCTCCTGCCATTGCGATA	*cps16D*	This study		400	
APP17HRM_for	GTAATGGCGGTGTAATGCTA	*cps17F*	This study	111	600	73 ± 0.1
APP17HRM_rev	AATGGCTGATGTTACTACAGTATT	*cps17F*	This study		600	
APP18HRM_for	TGGCAGCATAAAGGTCAATT	*cps18B*	This study	105	500	73.6 ± 0.1
APP18HRM_rev	ACGCTGTAAGTGTTTTGGTAT	*cps18B*	This study		500	
APP19HRM_for	ACGGCAAATAATCGAGTTACT	*cps19C*	This study	96	500	74.7 ± 0.2
APP19HRM_rev	AGCATCAGGATCAATGTCAAT	*cps19C*	This study		500	

Abbreviations: APP‐HRM, *Actinobacillus pleuropneumoniae*‐high resolution melting; PCR, polymerase chain reaction.

Nineteen reference strains (Table 1) were used to develop the HRM assays. Data analysis was performed using Rotor‐Gene Q Software 2.3.1 (Qiagen) by melting curve analysis as well as analysis of generated normalized and difference plots. Samples revealing typical melting curves above the threshold value of 0.5 *dF*/*dT* were considered positive.

To examine the intra‐ and interassay variability of the amplicon melting temperatures (*T*_m_) of the novel HRM assay (APP‐HRM1, APP‐HRM2, and APP‐HRM3) representing its repeatability, 1.25 ng of genomic DNA of all 19 serovar reference strains were tested in triplicates in three individual experiments.

### 2.4 Specificity

To determine the specificity of the HRM assay, an exclusivity panel of 27 bacterial isolates comprising 18 different bacterial strains was tested applying the three assays APP‐HRM1, APP‐HRM2, and APP‐HRM3.

### 2.5 Analytical sensitivity

To determine the analytical sensitivities of the HRM assay, all reference strains were examined. Given the genome size of *A. pleuropneumoniae* between 2.2 and 2.4 Mbp (Bossé et al., 2016; Foote et al., 2008; Xu et al., 2010; Zhan et al., 2010), one genome equivalent (GE) of each reference strain corresponded to approximately 2.5 fg of genomic DNA.

To analyze the range of detection and linearity of all 19 *A. pleuropneumoniae* serovars, a 10‐fold serial dilution series containing 12.5 ng (5 × 10^6^ GE), 1.25 ng (5 × 10^5^ GE), 125 pg (5 × 10^4^ GE), 12.5 pg (5 × 10^3^ GE), 1.25 pg (500 GE), 125 fg (50 GE), 12.5 fg (5 GE) of genomic DNA was tested in triplicates using APP‐HRM1, APP‐HRM2, and APP‐HRM3. The linearity was expressed by the correlation coefficient (*R* ^2^) for each of the 19 *A. pleuropneumoniae* serovars. To evaluate the limit of detection (LOD), the lowest dilution was determined, at which all triplicates showed a positive melting curve above a threshold value of 0.5 *dF*/*dT* and a standard deviation of *C*_*t*_ values ≤ 0.5 corresponding to a 95% confidence interval.

### 2.6 Efficiency

To calculate efficiencies of the HRM assays for each primer pair, *C_t_* values measured in triplicates were plotted against GE in form of standard curves using a 10‐fold dilution series (5 × 10^6^ GE, 5 × 10^5^ GE, 5 × 10^4^ GE, 5 × 10^3^ GE, 500 GE, 50 GE, and 5 GE) of genomic DNA of each serovar reference strain. The PCR efficiency (*E*) was calculated from the slope (*S*) of the dilution curve in the linear range using the following equation: *E* = (10^1/^ ^−S^−1) × 100.

### 2.7 Clinical isolates

Fifteen clinical isolates obtained from the routine diagnostic lab of the Section of Veterinary Bacteriology were tested using primer‐mixes for APP‐HRM1, APP‐HMR2, and APP‐HRM3. The *T*_m_ for each of the 15 isolates was determined and compared with the corresponding *T*_m_ obtained from the 19 *A. pleuropneumoniae* serovar reference strains.

## 3 RESULTS

### 3.1 Conditions of HRM assays

**Figure 1:**
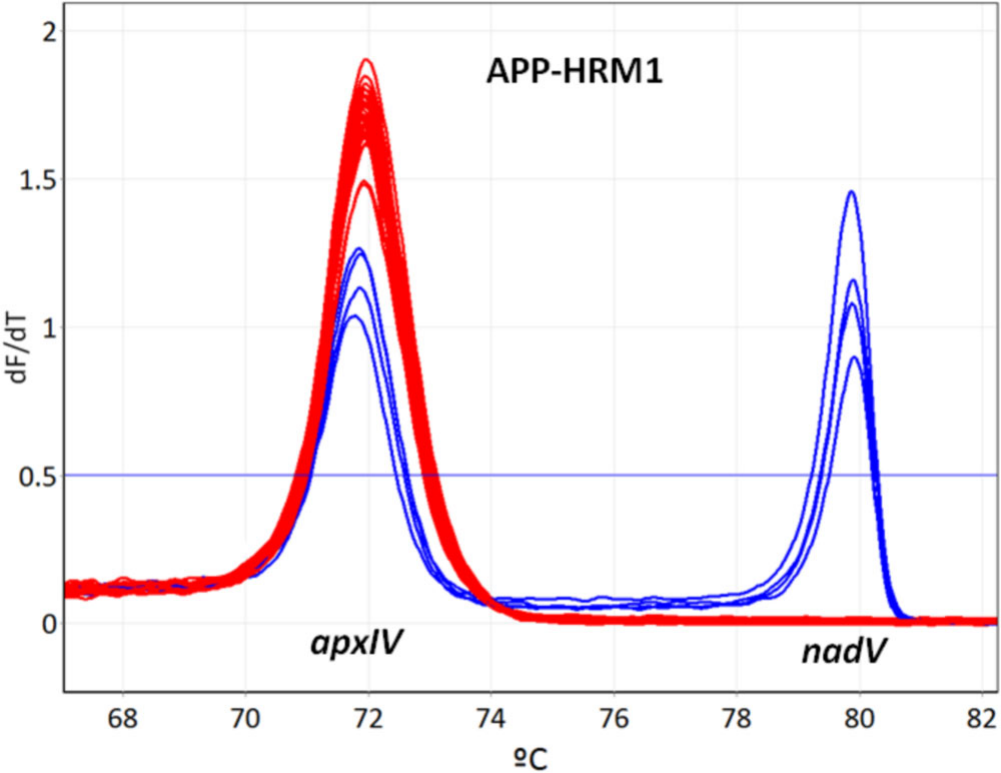
High resolution melting (HRM) for identification of *Actinobacillus pleuropneumoniae* and biovar 2. APP‐HRM1 assay allows targeting the species‐specific gene *apxIV* for identification of *A. pleuropneumoniae* and *nadV* for biovar 2 detection, respectively. *A. pleuropneumoniae* strains N273 (serovar reference strain 13), 3906 (serovar reference strain 14), SS4935 (serovar 2), and SS4936 (serovar 2) (represented in blue) all contain *apxIV* and *nadV*, whereas all remaining *A. pleuropneumoniae* strains tested in the study (represented in red) are biovar 1 and therefore only harbor *apxIV*

**Figure 2:**
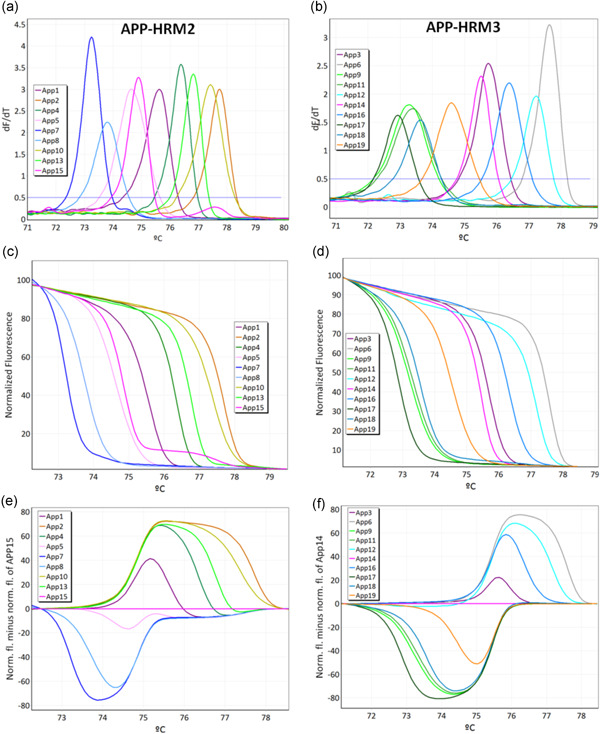
Illustration of high resolution (HRM) assay APP‐HRM2 (a–c) and APP‐HRM3 (d–f) capable of differentiating 19 serovars of *Actinobacillus pleuropneumoniae*. (a) Melting curves of the HRM step applying primers from APP‐HRM2. (b) Normalized plot for APP‐HRM2. (c) Difference plot for APP‐HRM2 normalized with DNA from *A. pleuropneumoniae* serovar 15. (d) Melting curves of the HRM step applying primers from APP‐HRM3. (e) Normalized plot for APP‐HRM3 (f) difference plot for APP‐HRM3 normalized with DNA from *A. pleuropneumoniae* serovar 14

**Figure 3:**
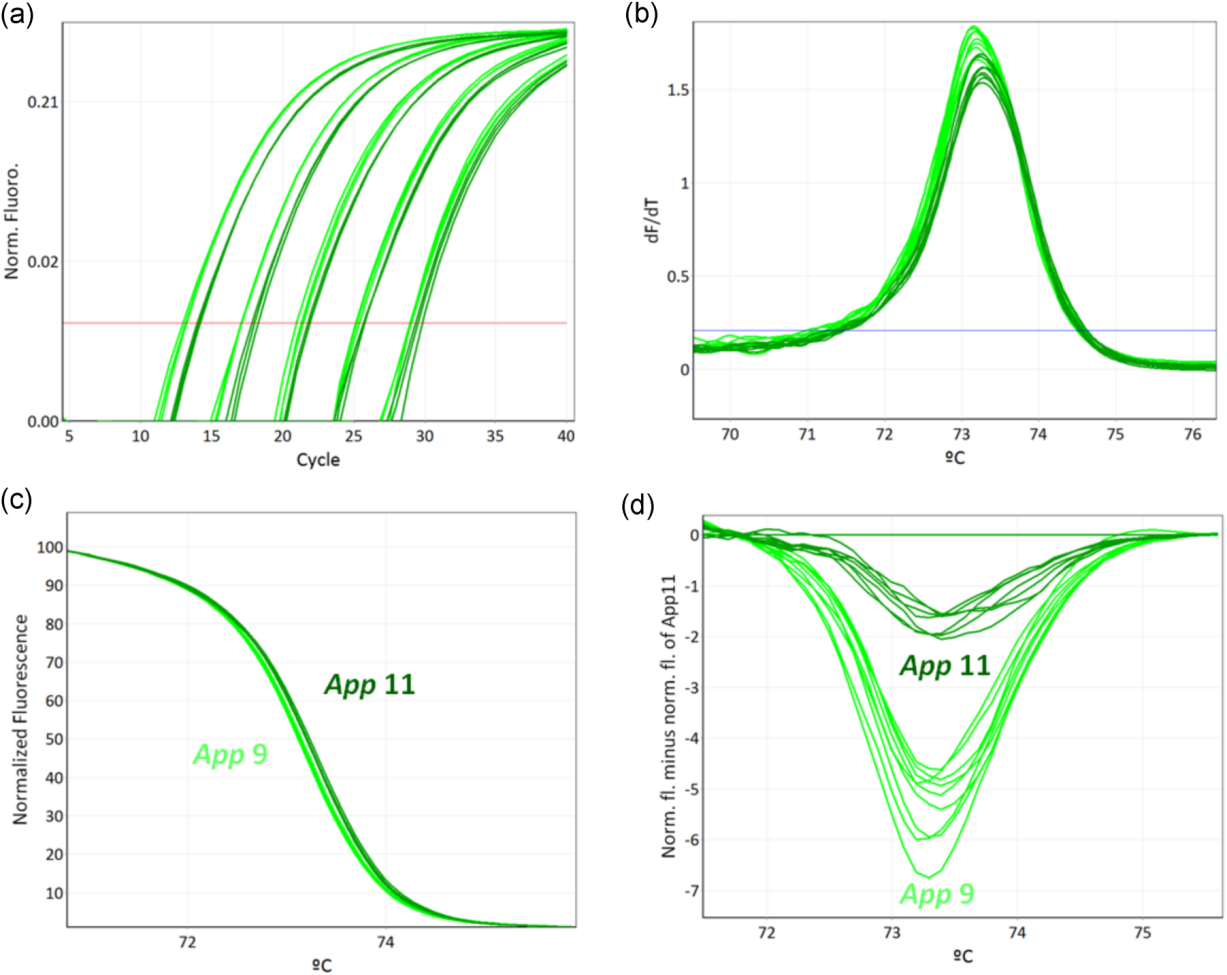
Identification of *Actinobacillus pleuropneumoniae* serovar 9 and serovar 11 using high resolution melting (HRM) assay APP‐HRM3. Differentiation of *A. pleuropneumoniae* serovar 9 and serovar 11 is based on a single‐nucleotide polymorphism in *cps9*/*11F*. A 10‐fold dilution series of *A. pleuropneumoniae* reference strains serovar 9 (light green) and serovar 11 (dark green) was tested in triplicates. Illustration of (a) PCR amplification curves, (b) melting curves of the HRM step, (c) normalized plot, and (d) difference plot normalized with genomic DNA from *A. pleuropneumoniae* serovar 11 (12.5 ng) visualizing two groups corresponding to *A. pleuropneumoniae* serovars 9 and 11. PCR, polymerase chain reaction

In a first step, APP‐HRM1 allows simultaneously screening of *A. pleuropneumoniae* positive isolates using *apxIV* toxin as species‐specific target and identification of biovar by targeting *nadV*. *A. pleuropneumoniae* strains N273 (serovar reference strain 13), 3906 (serovar reference strain 14), SS4935 (clinical isolate serovar 2), and SS4936 (clinical isolate serovar 2) all contain *apxIV* and *nadV*, whereas all remaining *A. pleuropneumoniae* strains tested in the study are biovar 1 and therefore only harbor *apxIV* (Figure 1). For serotyping purposes, all positive samples resulting from APP‐HRM1 were tested using two different primer mixes (APP‐HRM2 and APP‐HRM3) in parallel. The combination of the assays APP‐HRM2 and APP‐HRM3 is capable of differentiating all 19 serovars of *A. pleuropneumoniae* (Figure 2). Performing APP‐HRM3 using primers described in Table 5, serovar 9 and serovar 11 could be differentiated when represented as a difference plot (Figure 3).

The results of the variability assays from APP‐HRM1 resulted in a coefficient of variation of CV% ≤ 0.09% for the intra‐ and interassay variability when targeting *apxIV*, whereas *nadV* as a target yielded a coefficient of variation for the intra‐assay variation of CV% ≤ 0.03% and CV% ≤ 0.05% for the interassay variation, respectively (available at https://doi.org/10.5281/zenodo.6045373). APP‐HRM2 and APP‐HRM3 revealed an intra‐ and interassay variation coefficient of CV% ≤ 0.06%. The obtained low coefficient of variation for all three APP‐HRM assays of lower than 0.1% demonstrated the novel HRM to be highly reproducible and robust (available at https://doi.org/10.5281/zenodo.6045373).

### 3.2 Specificity

The tested exclusivity panel of 27 pathogenic bacteria resulted in negative results for all tested non‐*A. pleuropneumoniae* strains when performing APP‐HRM1, APP‐HRM2, and APP‐HRM3. Furthermore, the reference strains of all 19 *A. pleuropneumoniae* serovars did not cross‐react with other serovars (Figure 2). Hence, the novel HRM assay had a specificity of 100%.

### 3.3 Analytical sensitivity

**Figure 4:**
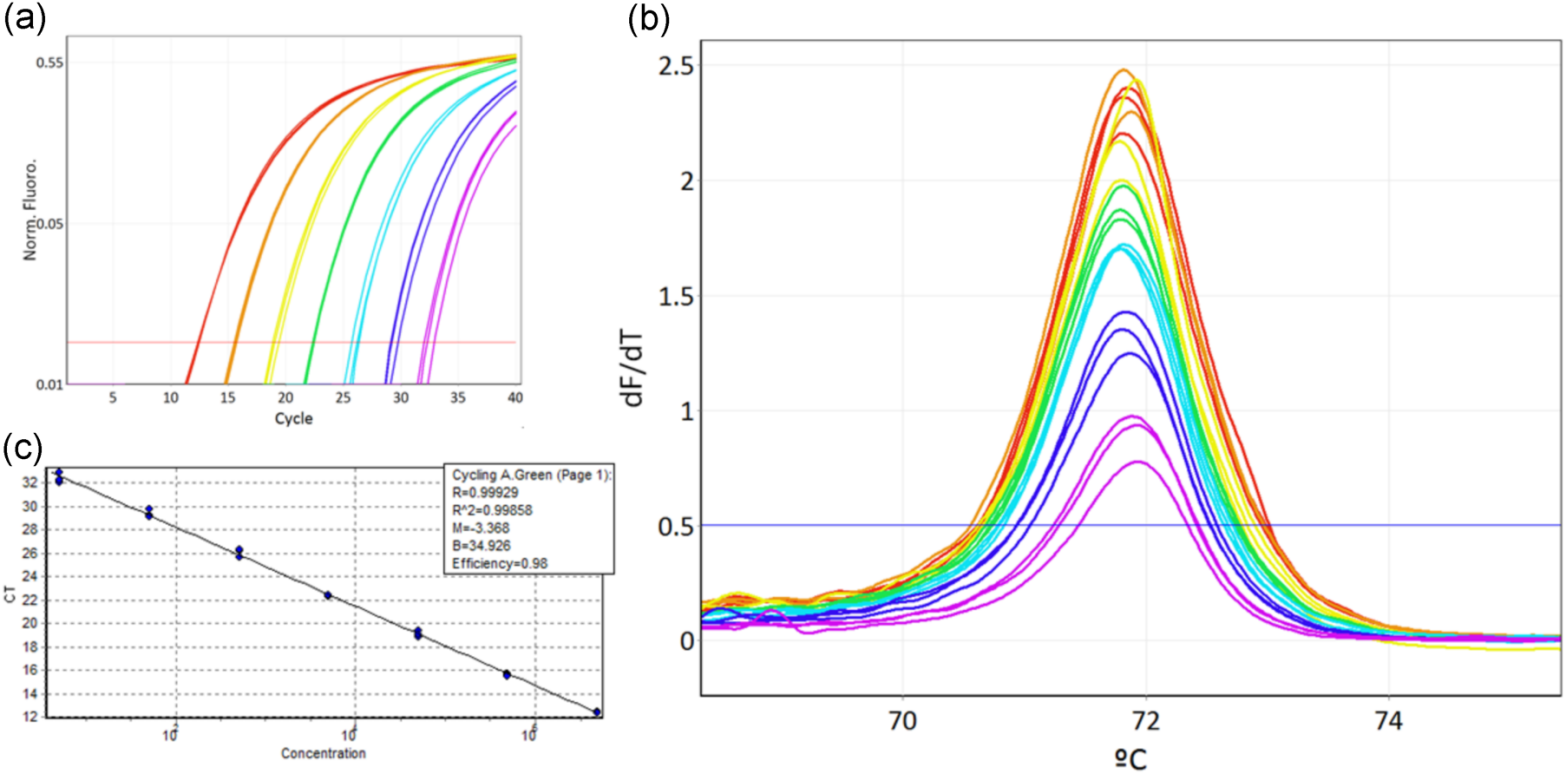
Identification of *Actinobacillus pleuropneumoniae* targeting *apxIV* (APP‐HRM1) illustrating high sensitivity. APP‐HRM1 was performed with an *A. pleuropneumoniae* tenfold dilution series of genomic DNA (here as an example *A. pleuropneumoniae* serovar 15) using primers targeting *apxIV*. DNA quantities of the tenfold dilution series were 5,000,000 genome equivalents (GE) (red), 500,000 GE (orange), 50,000 GE (yellow), 5000 GE (green), 500 GE (light blue), 50 GE (blue), and 5 GE (violet). Representation of the 10‐fold dilution series as (a) qPCR amplification plot; (b) melting curves of the HRM step illustrating a limit of detection of 5 GE; (c) standard curve indicating linearity across a large range of DNA quantities between 5,000,000 GE and 5 GE with a high correlation coefficient (*R* ^2^ > 0.99). HRM, high resolution melting; qPCR, quantitative polymerase chain reaction

**Figure A1:**
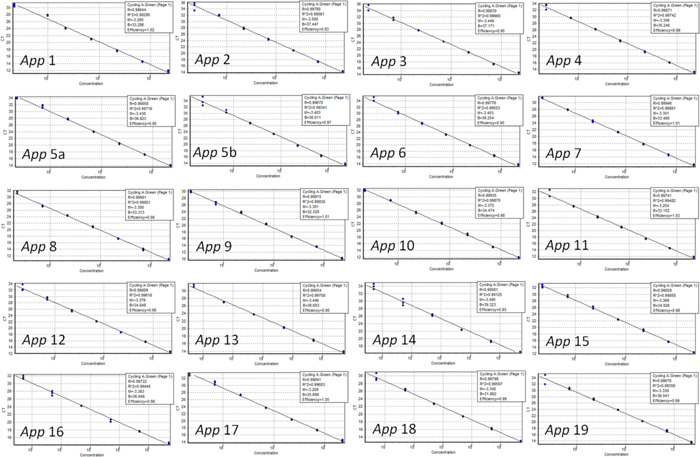
Standard curves of 10‐fold dilution series acquired by the serovar‐specific APP‐HRM1 assay in the dynamic range of 5 × 10^6^–5 genome equivalents for all reference *Actinobacillus pleuropneumoniae* strains are represented. PCR efficiencies between 93% and 103% with high correlation coefficients (*R* ^2^ > 0.99) were obtained. APP‐HRM, *Actinobacillus pleuropneumoniae*‐high resolution melting; PCR, polymerase chain reaction

**Figure A2:**
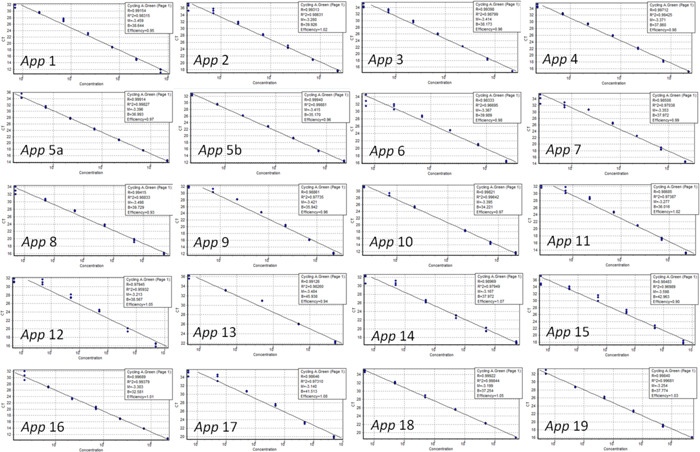
Standard curves of 10‐fold dilution series acquired by the serovar‐specific APP‐HRM2 and APP‐HRM3 assays in the range of linearity for all serovar reference strains of *Actinobacillus pleuropneumoniae* are represented. PCR efficiencies between 90% and 108% with correlation coefficients (*R* ^2^ > 0.96) were obtained. APP‐HRM, *Actinobacillus pleuropneumoniae*‐high resolution melting; PCR, polymerase chain reaction

Standard curves were obtained using *C*_t_ values from the tenfold dilution series of genomic DNA for each of the 19 *A. pleuropneumoniae* serovar reference strains amplified by *apxIV* and *nadV*‐specific primer pairs (APP‐HRM1) and by the 19 serovar‐specific primers pairs (APP‐HRM2 and APP‐HRM3) (Figures A1 and A2). For APP‐HRM1 the linear range of standard curves was between 5 × 10^6^ and 50 GE for all tested *A. pleuropneumoniae* reference strains. The following LODs were identified to be within the relevant confidence level of 95%: 5 GE for *A. pleuropneumoniae* serovar 1, 7, 8, 9, 10, 15, and 16; and 50 GE for *A. pleuropneumoniae* serovar 2, 3, 4, 5a, 5b, 6, 11, 12, 13, 14, 17, 18, and 19, respectively. The standard curves for APP‐HRM1 showed high correlation coefficients of *R* ^2^ > 0.99. For visualization, the high sensitivity obtained for all *A. pleuropneumoniae* serovars when performing APP‐HRM1, a representative dilution series of *A. pleuropneumoniae* serovar 15 is shown (Figure 4) highlighting a low LOD of 5 GE with linearity of the standard curve across a large range of DNA quantities between 5,000,000 GE and 5 GE.

For APP‐HRM2 and APP‐HRM3 the linear range of standard curves was more variable in contrast to APP‐HRM1 due to the increased complexity of the master mixes containing up to 10 *cps*‐specific primer‐pairs. Obtained LODs within the relevant confidence level of 95% were 5 GE for *A. pleuropneumoniae* serovar 5b; 50 GE for *A. pleuropneumoniae* serovar 4, 5a, 10, and 16; 500 GE for *A. pleuropneumoniae* serovar 1, 2, 3, 7, 8, 9, 11, 14, 18, and 19; and 5000 GE for *A. pleuropneumoniae* serovar 6, 12, 13, 15, and 17, respectively. Standard curves had correlation coefficients of *R* ^2^ > 0.96 (Table 6).

**Table 6:** Efficiency and limit of detection (LOD) of APP‐HRM1, APP‐HRM2, and APP‐HRM3 targeting *apxIV*, *nadV*, and serovar‐specific *cps* loci

*A. pleuropneumoniae* serovar	Strain	APP‐HRM1				APP‐HRM2, APP‐HRM3		
		LOD *apxIV*	LOD *nadV*	Efficiency (%)	*R* ^2^	LOD *cps*	efficiency	*R* ^2^
*App* serovar 1	ATCC 27088	5 GE		102	0.993	500 GE	95%	0.992
*App* serovar 2	P1875	50 GE		93	0.996	500 GE	102%	0.986
*App* serovar 3	ORG1224	50 GE		95	0.997	500 GE	96%	0.988
*App* serovar 4	M62	50 GE		99	0.999	50 GE	98%	0.994
*App* serovar 5a	K17	50 GE		95	0.997	50 GE	97%	0.998
*App* serovar 5b	L20	50 GE		97	0.993	5 GE	96%	0.999
*App* serovar 6	femø	50 GE		96	0.996	5000 GE	98%	0.967
*App* serovar 7	WF83	5 GE		101	0.999	500 GE	99%	0.970
*App* serovar 8	405	5 GE		98	0.998	500 GE	93%	0.988
*App* serovar 9	CVJ 13261	5 GE		101	0.998	500 GE	96%	0.977
*App* serovar 10	D13039	5 GE		98	0.999	50 GE	97%	0.996
*App* serovar 11	56153	50 GE		103	0.995	500 GE	102%	0.974
*App* serovar 12	8329	50 GE		98	0.996	5000 GE	105%	0.959
*App* serovar 13	N 273	50 GE	500 GE	95	0.997	5000 GE	94%	0.991
*App* serovar 14	3906	50 GE	500 GE	93	0.991	500 GE	107%	0.979
*App* serovar 15	HS143	5 GE		98	0.999	5000 GE	90%	0.970
*App* serovar 16	A‐85/14	5 GE		98	0.994	50 GE	101%	0.994
*App* serovar 17	16287‐1	50 GE		105	0.997	5000 GE	108%	0.973
*App* serovar 18	7311555	50 GE		99	0.996	500 GE	105%	0.998
*App* serovar 19	G1 9669	50 GE		99	0.994	500 GE	103%	0.997

*Note*: The LOD of APP‐HRM1 was between 5 and 50 genome equivalents (GE) corresponding to 12.5–125 fg of genomic DNA with PCR efficiencies of 93%–105%. LODs of APP‐HRM2 and APP‐HRM3 were between 5 and 5000 GE corresponding to 12.5 pg–12.5 fg genomic DNA with PCR efficiencies of 90%–108%.

Abbreviations: APP‐HRM, *Actinobacillus pleuropneumoniae*‐high resolution melting; LOD, limit of detection; PCR, polymerase chain reaction.

### 3.4 Efficiency

Using the equation mentioned in the methods, the efficiency values for each of the 19 *A. pleuropneumoniae* reference strains ranged between 93% and 105% for APP‐HRM1, whereas APP‐HRM2 and APP‐HRM3 revealed PCR efficiencies between 90% and 108% (Table 6, Figures A1 and A2).

### 3.5 Clinical isolates

**Figure 5:**
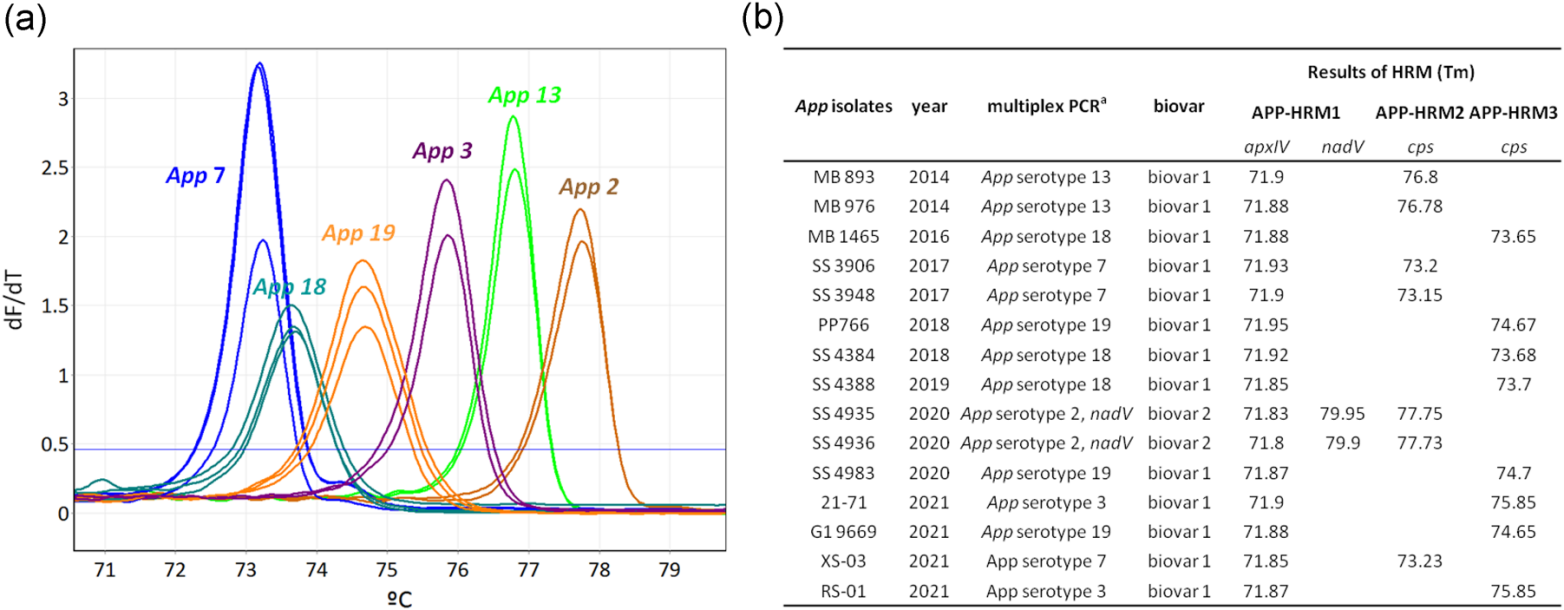
Representation of high resolution melting (HRM) results of DNA samples from 15 clinical *Actinobacillus pleuropneumoniae* isolates collected between 2014 and 2021 in Switzerland. (a) Illustration of HRM melting curves obtained with APP‐HRM2 and APP‐HRM3. (b) For each isolate, the corresponding amplicon melting temperatures (*T*_m_) obtained from APP‐HRM1, APP‐HRM2, and APP‐HRM3 are shown. ^a^Serovar determination by multiplex polymerase chain reaction (Bossé et al., 2018; Stringer et al., 2021)

Fifteen clinical isolates were analyzed with the novel HRM and the resulting Tms obtained from HRM assays APP‐HRM1, APP‐HRM2, and APP‐HRM3 were compared with *T*_m_ of the 19 *A. pleuropneumonia* serovar reference strains. All 15 clinical isolates could be identified as *A. pleuropneumoniae*, whereas serovar 2 strains SS 4935 and SS 4936 additionally demonstrated the presence of *nadV* thus corresponding to biovar 2. With the two serotyping HRM assays APP‐HRM2 and APP‐HRM3, all samples could be unambiguously assigned to the correct serovar by considering uniquely the *T*_m_ values (Figure 5).

## 4 DISCUSSION

*A. pleuropneumoniae* infection is a worldwide problem in the pig industry (Sassu et al., 2018). Referring to the severity of symptoms caused by *A. pleuropneumoniae* and based on the assumption of a high *A. pleuropneumoniae* prevalence (>0.3), Stygar and coworkers calculated the additional yearly costs between €0.4 and 24 per space unit (Stygar et al., 2016). Due to the challenges of antibiotic usage and the generation of antibiotic resistance, the most promising approach to preventing *A. pleuropneumoniae* infection lies in vaccination (Cao et al., 2020; Michael et al., 2015). Current market‐leading vaccines are based on inactivated Apx toxins and outer membrane components of *A. pleuropneumoniae* or inactivated *A. pleuropneumoniae* of selected serovars (Del Pozo Sacristán et al., 2014; Sipos et al., 2021). Especially for a vaccine targeting certain prevalent serovars, a good diagnostic tool, for fast and reliable identification of *A. pleuropneumoniae* serovars predominant in affected farms and/or regions, might be beneficial for fighting and eradicating the pathogen.

In the present study, an efficient molecular tool for the identification and serotyping of *A. pleuropneumoniae* was developed, demonstrating a robust and accurate assay. The novel HRM assay consisting of the species‐specific APP‐HRM1 and two serovar‐specific HRM assays (APP‐HRM2 and APP‐HRM3) demonstrated the specificity of 100% for all 19 known *A. pleuropneumoniae* serovars. Exclusivity testing showed full *A. pleuropneumoniae* specificity as no signal was detected in 18 different bacterial strains. Using APP‐HRM1, *A. pleuropneumoniae* can be detected very sensitively between a detection limit of 5–50 GE corresponding to 125 fg–1.25 pg of DNA, representing a high sensitivity allowing to detect even low levels of *A. pleuropneumoniae*‐infected tissues. In contrast, the serotyping assays APP‐HRM2 andAPP‐HRM3 did not reach the same sensitivity as APP‐HRM1 due to a much more complex composition of the primer mixes with up to 10 primer pairs used in the two serotyping assays. Importantly, new serovars could be identified by observation of an *apxIV*‐positive signal using APP‐HRM1 detecting *A. pleuropneumoniae* at the species level in combination with no corresponding serovar‐specific melting curve in APP‐HRM2 and APP‐HRM3.

The primer pairs targeting serovar 9 and 11 can differentiate the serovars since the amplified PCR product encompasses a single‐nucleotide polymorphism (SNP) in *cps* 9/11F leading to slightly different *T*_m_ of the corresponding PCR amplicons. Representation as a difference plot allows visualizing the slight difference in the melting curve. Due to missing clinical isolates representing these serovars, no validation using field isolates could be fulfilled. It is recommended in the future to test more isolates with serovar 9 and 11 of different origins for validation purposes and to prove the ability to robustly discriminate these closely related serovars.

When performing APP‐HRM1, PCR amplification of *apxIV* resulted in a melting curve with a *T*_m_ of 71.8 ± 0.2 unambiguously identifying *A. pleuropneumoniae*, while the melting curve obtained by *nadV* specific primers yielded *T*_m_ of 79.8 ± 0.2 determining biovar 2 isolates. Using APP‐HRM2 and APP‐HRM3, 13 *A. pleuropneumoniae* serovars (serovars 1, 4, 7, 8, 13, 16, 17, 18, and 19) could be determined explicitly inspecting the assigned *T*_m_ listed in Tables 3, 4, 5. In contrast, serovar 3 and 14, serovar 9 and 11, and serovar 5 and 15 cannot explicitly be distinguished uniquely from the *T*_m_. Serovar 3 (*T*_m_ = 75.7 ± 0.2) and serovar 14 (*T*_m_ = 75.5 ± 0.1), serovar 9 (73.3 ± 0.1) and serovar 11 (73.4 ± 0.1), serovar 5 (*T*_m_ = 74.5 ± 0.1) and serovar 15 (*T*_m_ = 74.8 ± 0.2), respectively, harbor partly overlapping *T*_m_. Additionally, serovars 2 (*T*_m_ = 77.6 ± 0.1) and 10 (*T*_m_ = 77.3 ± 0.1) and similarly, serovars 6 (*T*_m_ = 77.6 ± 0.1) and 12 (*T*_m_ = 77.2 ± 0.1) have close *T*_m_ values. Since this newly proposed HRM assay is based on the high resolution melting of PCR amplicons, which directly depends on its sequences, unique melting temperatures are expected for each serovar. It is a challenge to visualize the 19 different APP serovars in an HRM setting of only two reaction mixes. To unambiguously ensure the correct assignment of the serovar according to the Tm, it is recommended to perform the serotyping HRM assay (APP‐HRM2 and APP‐HRM3) using positive controls for each serovar.

Serovar prevalence differs from country to country. In England and Wales serovar 8 is most prevalent (Li et al., 2016), whereas serovar 7 plays an important role in Spain (Maldonado et al., 2009). Interestingly, several studies from central Europe reported clear dominance of serovar 2, as described in recent studies from Germany and Hungary (Sárközi et al., 2018; Schuwerk et al., 2021) and partially outdated ones from Belgium, Denmark, and the Netherlands (Dom et al., 1994; Jessing et al., 2003). Furthermore, in countries on other continents, such as Canada and Australia serovar 5, 7, and 15, respectively, were most frequently detected (Gottschalk & Lacouture, 2015; Turni et al., 2014). Serovar determination of 15 clinical *A. pleuropneumoniae* isolates collected between 2014 and 2021 in Switzerland revealed a quite heterogeneous serovar frequency with two to three isolates each of serovar 2, 3, 7, 13, 18, and 19. However, since a sample number of only 15 isolates is not representative, testing a larger strain collection in a future project would be necessary to determine the serovar prevalence and the accuracy of the HRM tool using clinical samples. It would be interesting to find out, whether one serovar is predominant, such as serovar 2 in neighboring countries, or if the serovar distribution of *A. pleuropneumoniae* in Switzerland is that heterogeneous.

To apply an HRM assay in the laboratory, a qPCR device capable of performing HRM is needed. Diverse brands of HRM‐compatible qPCR instruments exist in the market, which can be used for faster handling in comparison to conventional PCR. A closed one‐step system such as qPCR needs fewer manipulating steps in a shorter running time, in contrast to analyzing PCR products by agarose gel or capillary electrophoresis when working with conventional PCR. Therefore, the HRM method does not require any downstream processing of samples after qPCR thus increasing its efficiency, data is easy to access and interpret compared to the conventional methodology using band pattern recognition.

The development of the HRM assay represents a molecular tool, which allows screening for *A. pleuropneumoniae*. In an upcoming project, which aims at monitoring the continuance of *A. pleuropneumoniae* in Switzerland, further DNA samples directly isolated from lung and tonsillar tissue will be tested and validated systematically. Some preliminary experiments revealed the correct serovar assignment of diagnostic DNA samples of *A. pleuropneumoniae* positive animals, suggesting that clinical samples might be detected when performing the novel HRM assay.

## 5 CONCLUSION

From a monitoring perspective, as well as due to the different courses of disease associated with the various serovars, it is essential to differentiate the *A. pleuropneumoniae* serovars in different herds or countries. The developed species‐specific HRM assay (APP‐HRM1), as well as the two serotyping HRM assays distinguishing between all 19 serovars of *A. pleuropneumoniae* (APP‐HRM2 and APP‐HRM3), provide a useful diagnostic tool to discover virulent serovars. In the future, this newly proposed HRM assay may disclose the possibility for screening clinical samples and further evaluating the assay using a larger strain collection. Such a molecular tool can be applied in routine veterinary laboratories to get a rapid and precise overview of *A. pleuropneumoniae* strains presently circulating among pig farms.

Knowing the prevalent serovar, the right vaccines can be administered and pigs carrying virulent *A. pleuropneumoniae* strains can be prevented from being introduced into farms without any history of *A. pleuropneumoniae*‐related disease. Moreover, applying this HRM approach using the characteristic melting patterns of amplicons allows to potentially identify new *A. pleuropneumoniae* serovars in a straightforward and efficient way, thereby improving the presently available diagnostic tools.

## CONFLICTS OF INTEREST

The authors declare no conflicts of interest.

## ETHICS STATEMENT

None required.

## AUTHOR CONTRIBUTIONS

**Simone Scherrer**: Conceptualization‐lead, investigation‐lead, writing original draft‐lead. Sophie Peterhans: Investigation‐supporting, methodology‐lead, writing original draft‐supporting. **Christine Neupert**: Investigation‐supporting, resources‐equal, writing‐review & editing‐supporting. **Fenja Rademacher**: Investigation‐supporting. **Giody Bartolomei**: Investigation‐supporting, writing‐review & editing‐supporting. **Xaver Sidler**: Resources‐equal, writing‐review & editing‐supporting. **Roger Stephan**: Project administration‐lead, writing‐review & editing‐supporting.

## Data Availability

All data are provided in this article except for the supplemental data, which are available in the Zenodo repository at https://doi.org/10.5281/zenodo.6045373 (Table S1: Inter‐ and intra‐assay variability of APP‐HRM1 and Table S2: Inter‐ and intra‐assay variability of APP‐HRM2 and APP‐HRM3).

## 1

Figures A1 and A2.
